# Dr. Kenneth A. Jacobson: First Winner of the Tu Youyou Award, in Honor of the Co-Recipient of the 2015 Nobel Prize in Physiology or Medicine

**DOI:** 10.3390/molecules21121656

**Published:** 2016-12-01

**Authors:** Derek J. McPhee

**Affiliations:** Editor-in-Chief of *Molecules*; Senior Director, Technology Strategy, Amyris, Inc., 5885 Hollis St, Suite 100, Emeryville, CA 94608, USA; mcphee@mdpi.com

On behalf of MDPI, the journal *Molecules*, its Editorial Board and the award selection committee, whose diligent work evaluating the more than 50 outstanding quality nominations received for the award is gratefully acknowledged, it is with great pleasure that I announce that the winner of the 2016 TU YOUYOU AWARD, sponsored by MDPI, is Dr. KENNETH A. JACOBSON, currently the Section Chief of the Molecular Recognition Section of the Laboratory of Bioorganic Chemistry (LBC) and Lab Chief of the LBC at the National Institute of Diabetes and Digestive and Kidney Diseases (NIDDK) of the National Institutes of Health in Bethesda, MD, USA. As the awardee, Dr. Jacobsen will receive an honorarium of 3000 CHF and an engraved plaque.

Described by his peers as “the leading medicinal chemist in the world, producing a remarkable number of selective agonists and antagonists, as well as radioligands and photoaffinity labels for both P1 (adenosine) and P2Y (ATP, UTP and UDP) G protein-coupled receptor (GPCR) subtypes”, new drugs developed under his direction in a government lab of modest size and resources compared to the pharma industry are currently being developed primarily for therapeutic applications, for example for cancer and autoimmune inflammatory diseases, such as rheumatoid arthritis and psoriasis, but also for important unmet medical needs, such as chronic neuropathic pain, which affects a large fraction of the population. In addition, over three dozen chemical probes introduced by the Jacobson lab are available commercially as research tools and are used widely for the study of purine receptors, other GPCRs and ion channels

In light of these and his many other accomplishments, there can be no doubt that Dr. Jacobson more than meets the award criteria of groundbreaking research and significant contributions to the advancement of the natural product and medicinal chemistry field, and we are honored to add this modest award to his many others in the fields of chemistry and pharmacology.

